# Enoxaparin for the prevention of preeclampsia and intrauterine growth restriction in women with a prior history – an open-label randomised trial (the EPPI trial): study protocol

**DOI:** 10.1186/s12884-016-1162-y

**Published:** 2016-11-22

**Authors:** K. M. Groom, L. M. McCowan, P. R. Stone, L. C. Chamley, C. McLintock, the EPPI trial Study Group

**Affiliations:** 1 Department of Obstetrics and Gynaecology, Faculty of Medical Health Sciences, University of Auckland, Private Bag 92019, Auckland, New Zealand; 2 National Women’s Health, Auckland City Hospital, Private Bag 92024, Auckland, New Zealand

**Keywords:** Preeclampsia, Intrauterine growth restriction, Fetal growth restriction, IUGR, Small for gestational age, Enoxaparin, Low molecular weight heparin, Randomised trial

## Abstract

**Background:**

Preeclampsia and intrauterine fetal growth restriction (IUGR) are two of the most common causes of maternal and perinatal morbidity and mortality. Current methods of predicting those at most risk of these conditions remain relatively poor, and in clinical practice past obstetric history remains the most commonly used tool. Aspirin and, in women at risk of preeclampsia only, calcium have been demonstrated to have a modest effect on risk reduction. Several observational studies and randomised trials suggest that low molecular weight heparin (LMWH) therapy may confer some benefit.

**Methods/design:**

This is a multicentre open label randomised controlled trial to determine the effect of the LMWH, enoxaparin, on the prevention of recurrence of preeclampsia and/or IUGR in women at high risk due to their past obstetric history in addition to standard high risk care for all participants.

Inclusion criteria: A singleton pregnancy >6^+0^ and <16^+0^ weeks gestation with most recent prior pregnancy with duration >12 weeks having; (1) preeclampsia delivered <36^+0^ weeks, (2) Small for gestational age (SGA) infant <10^th^ customised birthweight centile delivered <36^+0^ weeks or, (3) SGA infant ≤3^rd^ customised birthweight centile delivered at any gestation. Randomisation is stratified for maternal thrombophilia status and women are randomly assigned to ‘standard high risk care’ or ‘standard high risk care’ plus enoxaparin 40 mg from recruitment until 36^+0^ weeks or delivery, whichever occurs sooner. Standard high risk care includes the use of aspirin 100 mg daily and calcium 1000–1500 mg daily (unless only had previous SGA with no preeclampsia).

The primary outcome is preeclampsia and/or SGA <5^th^ customised birthweight centile. Analysis will be by intention to treat.

**Discussion:**

The EPPI trial has more focussed and clinically relevant inclusion criteria than other randomised trials with a more restricted composite primary outcome. The inclusion of standard use of aspirin (and calcium) for all participants will help to ensure that any differences observed in outcome are likely to be related to enoxaparin use. These data will make a significant contribution to future meta-analyses and systematic reviews on the use of LMWH for the prevention of placental mediated conditions.

**Trial registration:**

ACTRN12609000699268 Australian New Zealand Clinical Trials Registry. Date registered 13/Aug/2009 (prospective registration).

## Background

Preeclampsia and intrauterine fetal growth restriction (IUGR) are two of the most common causes of maternal and perinatal morbidity and mortality. Preeclampsia complicates approximately 3–5% of pregnancies and remains one of the most common causes of direct maternal death in the developed world [1]. IUGR is more difficult to define and measure but approximately 10% of all infants will be born small for gestational (SGA) defined as birthweight < 10^th^ customised birthweight centile and at least two thirds of these have evidence of abnormal uterine and umbilical Doppler waveforms if diagnosed prior to birth suggesting significant utero-placental disease and fetal growth restriction [2]. Infants who are born growth restricted with or without maternal preeclampsia are at increased risk of morbidity and mortality during the perinatal period [3–5], and this risk continues into childhood and adult life with higher rates of neurosensory disability, cognitive impairment, short stature, hypertension, diabetes and long term cardiovascular disease [6–11]. Women with preeclampsia and/or IUGR fetuses are more likely to require preterm delivery, with over one third of women with preeclampsia and 15% of SGA infants delivering <37 weeks [12]. Iatrogenic preterm birth often results in operative delivery and therefore has inherent risks to the mother and results in the significant additional risks of prematurity for an already compromised infant [3, 13–15].

There are a wide variety of identified risk factors for preeclampsia and/or IUGR but few contribute significantly to risk prediction models which at best remain modest [16, 17]. Until relatively recently it has been suggested that inherited thrombophilias are associated with preeclampsia and IUGR, however, more recent evidence from prospective cohort studies suggests this association, if present, is only weak [18]. Past obstetric history is the most commonly used method for risk assessment in current clinical practice. Women who have had previous preeclampsia and/or IUGR are at significant risk of recurrence especially when the disease occurred early in the previous pregnancy with rates ranging from 15 to 47% depending on the severity of previous disease [19–22].

Both conditions are considered placental diseases and there are likely synergies in pathological mechanisms which may present as a maternal syndrome (preeclampsia only), a fetal syndrome (IUGR only) or a combined clinical syndrome (preeclampsia and IUGR) depending on fetal and maternal responses to the pathological process. The synergies in the pathological mechanisms have led many investigators to research common therapeutic and preventative strategies for both diseases. Aspirin and calcium have been trialled in a large number of randomised studies in a variety of populations and although effect sizes are modest both significantly reduce the incidence of preeclampsia [23], and aspirin also decreases the incidence of SGA [24]. They should be considered as standard practice in women at high risk of these conditions [25].

Heparin and low molecular weight heparin (LMWH) have also been proposed as potential preventative therapies for a number of placental mediated pregnancy complications including preeclampsia and IUGR. Their effect may relate to their anticoagulant effect although it is also likely that additional effects on trophoblast development may be more significant [26–28]. Initial interest in the use of heparin for the prevention of placental mediated complications centred on the treatment of recurrent miscarriage associated with anti-phospholipid syndrome (APS). Early studies demonstrated significant benefit of unfractionated heparin with aspirin when compared to aspirin alone [29, 30]. This led many clinicians to routinely treat these women with LMWH for all pregnancy complications associated with APS and this practice has continued despite subsequent studies failing to demonstrate the same improvements in pregnancy outcomes with LMWH and aspirin [31, 32].

Some clinicians extended their use of heparin and LMWH for the prevention of preeclampsia and IUGR to women regarded at high risk of these complications for other reasons, such as those with renal disease [33], women with ACE DD polymorphism [34] and women with inherited thrombophilia [35, 36]. These small observational and non-placebo controlled trials suggested significant benefits associated with heparin and LMWH.

Subsequently a number of randomised controlled trials have been reported. Initial trials focussed specifically on populations with or without thrombophilia, believing that the risk of these placental diseases may be significantly different between the groups and may respond differently to intervention with heparin or LMWH. In 2009, Rey et al. reported the outcomes of a randomised trial of LMWH in women with a past history of placental mediated problems (including preeclampsia and IUGR) and no thrombophilia. This trial demonstrated a large reduction in the primary composite outcome (severe preeclampsia, birthweight <5th centile, major abruption or fetal death after 20 weeks) 5.5 vs 23.6% adjOR 0.15, 95% CI 0.03–0.70 [37]. However the study was halted after just over one third of the planned participants had been recruited (results of 110 participants analysed of a planned sample size of 276 women). The FRUIT-RCT reported similar promising outcomes associated with LMWH use for women with inherited thrombophilia, demonstrating a significant reduction in the recurrence of hypertensive disease (preeclampsia, HELLP syndrome and/or eclampsia) <34 weeks gestation, 8.7 vs 0% risk difference 8.7, 95% CI 1.9–15.5%) [38]. This study also did not complete recruitment of initial planned sample size (139 of 262).

Subsequent published trials including the ‘Heparin in pregnant women with adverse pregnancy outcome to improve rate of successful pregnancy’ (HAPPY) Study [39], the ‘Prevention of maternal and perinatal complications by enoxaparin in women with previous severe preeclampsia’ (HEPEPE) Study [40] and the ‘Thrombophilia in Pregnancy Prophylaxis Study’ (TIPPS Study) [41] have failed to demonstrate any improvement in outcome with LMWH.

All of these trials have had prolonged recruitment phases (up to 12 years [41]) and have been difficult to recruit to. Several trials have stopped early due to recruitment difficulties and for perceived overwhelming effect [37] or futility after interim data analysis [39]. Of note, these previous randomised controlled trials have had broad inclusion criteria and diverse composite outcome measures.

The EPPI trial aims to be more precise, and clinically relevant, with its inclusion criteria and primary outcome measures specific to women at high risk of preeclampsia and/or IUGR. Account will be made of each participant’s thrombophilia status but this will not define the study population. Standard high risk care will include the use of aspirin and, where appropriate, calcium for all women. This is the first trial in this setting to also include serial assessment of placental and angiogenic growth factors.

## Methods/design

### Aim

To assess the effectiveness of LMWH, enoxaparin 40 mg daily, for the prevention of recurrence of preeclampsia and IUGR regardless of thrombophilia status and in addition to standard high risk antenatal care (including the use of aspirin and, where appropriate calcium).

### Study design

A multicentre open label randomised controlled trial.

### Study setting

The trial will recruit women from high risk clinic settings in New Zealand at National Women’s Health, Auckland City Hospital; in Australia at the Royal Women’s Hospital, Melbourne, the Mercy Hospital for Women, Melbourne and the Sunshine Hospital, Melbourne; and in the Netherlands at the Academic Medical Centre, Amsterdam.

### Inclusion criteria

Women are eligible for the trial if they are >6^+0^ and <16^+0^ weeks gestation with fetal viability and a singleton pregnancy confirmed on ultrasound scan and at risk of preeclampsia and/or IUGR based on their past obstetric history. This is defined by (1) previous preeclampsia delivered <36^+0^ weeks in their last on-going pregnancy reaching >12 weeks, (2) previous SGA < 10^th^ customised birthweight centile delivered <36^+0^ weeks in their last ongoing pregnancy reaching >12 weeks with no major fetal anomaly or, (3) previous SGA ≤ 3^rd^ customised birthweight centile delivered at any gestation in their last on-going pregnancy reaching >12 weeks with no major fetal anomaly.

### Exclusion criteria

Women are not eligible for the trial if they have (1) any contraindication to LMWH use, (2) a requirement for LMWH use such as previous thrombosis, APS, (3) previous successful pregnancy with LMWH treatment, (4) multiple pregnancy, (5) known pre-existing type 1 or 2 diabetes or renal disease (with serum creatinine >150), (6) thrombocytopenia (platelet count <80 × 10^9^/L) prior to randomisation or (7) a known major fetal anomaly/chromosomal abnormality.

### Trial entry and informed consent

All eligible women will be invited to participate in the study. Trial information will be provided verbally and by a written information sheet by clinicians caring for women and research midwives. Eligible women will be encouraged to take time to consider involvement in the study and to discuss participation with their partners and/or family prior to providing written consent. For women who decline to participate in the trial, antenatal care relevant to their risk factors will be offered, the use of LMWH will not be offered as part of standard care. All trial participants will be free to withdraw from the study at any time if they so wish, appropriate on-going antenatal care will be arranged according to clinical risk.

### Randomisation

A computer generated randomisation programme will be used with stratification for recruiting site and inherited thrombophilia status to ensure equal distribution across study groups. Women who have been tested prior to trial involvement will be assigned to (i) positive thrombophilia or (ii) negative thrombophilia. For women not tested or partially tested (with negative result), samples will be taken before any use of LMWH and tested for antithrombin deficiency, protein C deficiency, protein S deficiency, factor V Leiden, and the prothrombin gene mutation. Results will not be revealed to participant, clinicians or investigators until the study is completed. These women will be assigned to (iii) unknown thrombophilia status. Randomisation will be in blocks of five.

All participants will be assigned a sequential study identifying number (ID) according to thrombophilia status. At the lead recruiting site (National Women’s Health, Auckland City Hospital) randomisation will occur by telephone to the hospital pharmacy clinical trials service and study group allocation made by study ID. In all other sites sequential sealed opaque envelopes for each study ID will be opened to reveal study group.

### Study groups

Women will be assigned to one of two groups; Group one will receive ‘standard high risk care’ and Group two will receive ‘standard high risk care’ and enoxaparin 40 mg sc (Clexane®, Sanofi-Aventis) [42] daily from recruitment (at gestational age >6^+0^ and <16^+0^ weeks) until 36^+0^ weeks or delivery, whichever occurs sooner.

Standard high risk care is defined as care co-ordinated by a high risk antenatal clinic service, aspirin 100 mg daily until 36^+0^ weeks and calcium 1000–1500 mg daily until 36^+0^ weeks (unless only had previous SGA with no preeclampsia as eligibility criteria). This is an open-label randomised study and all participants, clinicians and investigators will be aware of study group assignment.

### Obstetric care and trial procedure (Fig. 1)

**Fig. 1:**
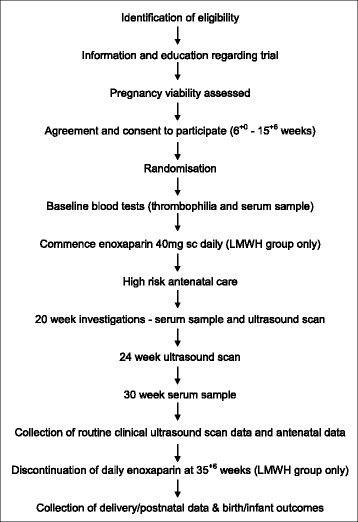
Individual participant trial schedule

All women will have serum samples taken at recruitment, 20 and 30 weeks gestation. Samples will be centrifuged and stored in −80 °C freezer for later assessment of placental and angiogenic growth factors (Table 1). At the time of fetal anatomy scan, standard fetal growth parameters [43, 44] and uterine and umbilical arterial Doppler waveforms [45] will be recorded. These ultrasound measurements will be repeated at 24 weeks’ gestation. Data from any additional antenatal ultrasound scans will also be collected.

**Table 1:** EPPI trial key secondary study outcomes

Combined	Preeclampsia and/or SGA <10th centile Preeclampsia and/or SGA <10th centile delivered <35^+6^ weeks Preeclampsia and/or SGA <10th centile delivered <33^+6^ weeks Preeclampsia and SGA <10^th^ centile delivered at any gestation
Maternal	Preeclampsia Severe preeclampsia Placental abruption Caesarean section delivery Antepartum haemorrhage Postpartum haemorrhage <500mls and <1000mls
Infant	SGA <10^th^ centile SGA <5^th^ centile SGA <3^rd^ centile Unexplained intrauterine fetal death Stillbirth Gestational age at delivery Mean birthweight Mean birthweight centile Neonatal Intensive Care Unit admission Neonatal death
Uteroplacental	Abnormal uterine artery Doppler waveform at 20 or 24 weeks Abnormal umbilical artery Doppler waveform at 20 or 24 weeks
Placental & angiogenic growth factors	soluble fms-like tyrosine kinase-1 (sFlt-1) soluble endoglin (soluble Eng) Endothelin-1 (ET-1) Placental growth factor (PLGF) soluble vascular cell adhesion molecule 1 (VCAM1)

*SGA* small for gestational age, all SGA measured by customised birthweight centile [46]

For women receiving LMWH, an educational session regarding injection technique will be provided by a research midwife prior to treatment commencement. LMWH injections will be re-supplied on a monthly basis from hospital pharmacies. Treatment will continue until 36^+0^ weeks but additional safety indications to stop treatment sooner include; need for delivery prior to 36^+0^ weeks (stop once decision made for delivery or 12 h prior to induction of labour or elective caesarean section, whichever is later), episode of threatened preterm labour (treatment can be recommenced if symptoms settle), clinical evidence of placental abruption or thrombocytopenia with platelet count <80 × 10^9^/L.

Antenatal, intra-partum and postnatal care will be provided by the local obstetric team caring for each woman. Pregnancy, labour, postnatal and neonatal data will be collected by research staff from maternal and infant clinical records up to the time of hospital discharge.

### Study outcomes

The primary outcome is the incidence of preeclampsia and/or small for gestational age (SGA) <5^th^ customised birthweight centile [46]. Preeclampsia will be defined as new onset hypertension (systolic BP ≥140 mmHg and/or diastolic BP ≥90 mmHg) arising after 20 weeks gestation with evidence of significant proteinuria (dipstick proteinuria (≥1+) subsequently confirmed by spot urine protein/creatinine ratio ≥ 30 mg/mmol and/or 24 h urine protein excretion >0.3 g) or any multi-system complication including haematological, liver, renal and neurological involvement [47]. IUGR is difficult to define and measure and so SGA defined by customised birthweight centiles will be used as the most reproducible surrogate. Customised centiles adjusting for infant sex, gestation at delivery and maternal variables—parity, ethnicity, height and weight will be used [46]. The key secondary study outcomes are shown in Table 1.

### Sample size

We have estimated a risk of recurrence of preeclampsia and/or SGA < 5^th^ customised centile for the included population of 25% when managed routinely with aspirin (and calcium). A trial of 160 participants, allowing for a 5% drop-out/early miscarriage rate will have 80% power at a two-sided significance level of 0.05 to detect a difference between 25 and 7% [37].

### Data monitoring committee (DMC) and interim analysis

An independent DMC comprising of a statistician, obstetrician, and obstetric physician with clinical trials experience will review serious adverse events. They will review the results of an interim analysis once 98 participants have been recruited and completed pregnancy. A study group of 49 participants in each arm will have 80% power at a two-sided significance level of 0.05 to detect a difference between 25 and 4%. The DMC will report to the trial investigator group and may advise discontinuation of the trial if there is an excess of serious adverse events in the LMWH treatment group or if a significant benefit is already demonstrated. Results of the interim analysis will only be known to members of the DMC and the trial investigator group. Responsibility for early discontinuation of the trial will ultimately rest with the trial investigator group.

### Data analysis

All data will be collected and stored under de-identified study ID. Data will be analysed by an independent statistician. Population characteristics between the groups will be compared initially to identify any potential confounding variables. Data will be analysed on an intention to treat basis comparing primary and secondary outcomes in both groups. Analyses will make adjustment for recruiting site and thrombophilia status. *P*-value <0.05 will be considered significant.

## Discussion

This is a protocol for a randomised trial to assess the role of the LMWH, enoxaparin, to prevent recurrence of preeclampsia and IUGR in women at high risk based on their past obstetric history. The EPPI trial has been designed to focus specifically on the related conditions of preeclampsia and IUGR. The inclusion criteria aim to identify women with or without thrombophilia but do not include less specific or poorly defined conditions such as past venous thromboembolism, recurrent miscarriage, unexplained fetal death and placental abruption as seen in some other trials of heparin therapy for the prevention of preeclampsia and IUGR. The pathological mechanisms of such a wide variety of conditions are likely to be significantly different from those of preeclampsia and IUGR and a single preventative therapy less likely to be effective for all. Similarly the composite primary outcome measure for this study has been limited to preeclampsia and a measure of IUGR (SGA) so results are more likely to be relevant to these placental mediated conditions.

Another strength of this study is that both treatment arms will receive standard high risk care including the use of aspirin in all women and, for those with previous history of preeclampsia, the use of calcium. Any differences observed in outcome are likely to be related to enoxaparin use.

This study will include 160 women and is one of the larger studies in this area. The findings of this trial will be available for future meta-analysis and systematic reviews. It will significantly contribute to and further current knowledge regarding the use enoxaparin in pregnancy to reduce recurrence of preeclampsia and IUGR.
